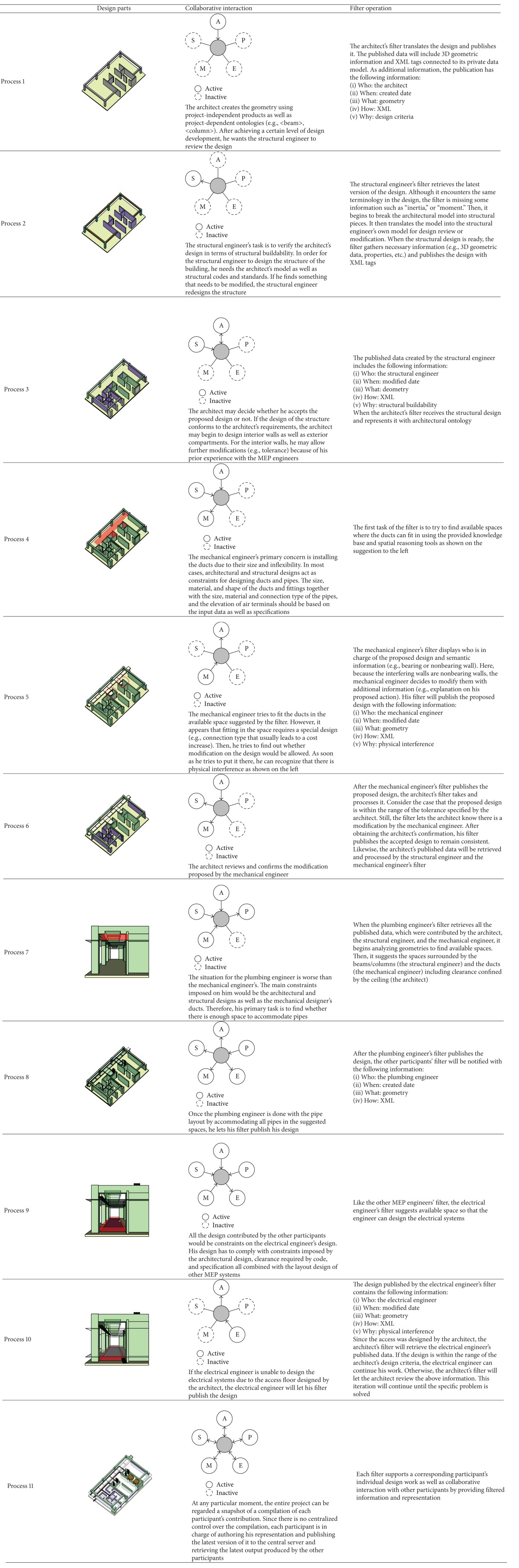# A Filter-Mediated Communication Model for Design Collaboration in Building Construction

**DOI:** 10.1155/2014/808613

**Published:** 2014-09-17

**Authors:** Jaewook Lee, Yongwook Jeong, Minho Oh, Seung Wan Hong

**Affiliations:** ^1^Department of Architectural Engineering, Sejong University, 98 Gunja-Dong, Gwangjin-Gu, Seoul 143-747, Republic of Korea; ^2^Emerging Tech. Lab, Samsung SDS, 9th Floor, ASEM Tower, Samsung-Dong, Gangnam-Gu, Seoul 135-798, Republic of Korea; ^3^Faculty of Architecture and Town Planning, Technion-Israel Institute of Technology, Technion City, 32000 Haifa, Israel

## Abstract

Multidisciplinary collaboration is an important aspect of modern engineering activities, arising from the growing complexity of artifacts whose design and construction require knowledge and skills that exceed the capacities of any one professional. However, current collaboration in the architecture, engineering, and construction industries often fails due to lack of shared understanding between different participants and limitations of their supporting tools. To achieve a high level of shared understanding, this study proposes a filter-mediated communication model. In the proposed model, participants retain their own data in the form most appropriate for their needs with domain-specific filters that transform the neutral representations into semantically rich ones, as needed by the participants. Conversely, the filters can translate semantically rich, domain-specific data into a neutral representation that can be accessed by other domain-specific filters. To validate the feasibility of the proposed model, we computationally implement the filter mechanism and apply it to a hypothetical test case. The result acknowledges that the filter mechanism can let the participants know ahead of time what will be the implications of their proposed actions, as seen from other participants' points of view.

## 1. Introduction

The success of collaboration depends on whether the participants achieve shared understanding [1]. The fragmentation of the architecture, engineering, and construction (AEC) industries renders this issue more critical. Although achieving and sustaining shared understanding are difficult, it is a key ingredient of creative collaboration. Shared understanding allows each of the participants to comprehend, critique, debate, adopt, or incorporate the propositions made by the other participants into an emerging collective creation [2]. For achieving shared understanding in the AEC industry, it has been regarded as a promising solution to increase interoperability among different CAD systems by way of organizing efficient databases [3–8].

The initial effort started with standardizing product descriptions including geometric information and constructing databases to organize them. Following the standardization of product model data, Eastman et al. [9] proposed engineering data model (EDM) to manage heterogeneous information carried by different design and engineering applications. With these motivations, building information modeling (BIM) has emerged as a key technology for collaboration and has been widely adopted in the AEC industry [10], which is the approach of centralized data models such as ISO 10303 standard for the exchange of product model data (STEP), CIMsteel integration standard version 2 (CIS/2), and industry foundation classes (IFC).

The underlying theoretical assumption of these efforts is that a building is a product composed of heterogeneous products. This assumption has been relatively valid and even successfully realized in several related industries, such as the automotive and shipbuilding manufacturing industries. However, the building and construction industry continues to lag behind in this development [11].

### 1.1. Capabilities of Current BIM Tools and IFCs

One of the leading BIM solutions available today is the Revit platform [12]. Revit started with a standalone and parametric building modeler based on database technology for architecture design that integrates views, components, parametric relationships, and annotations into a fully coordinated and consistent building information model. Recently, rather than standalone software, Revit has evolved to Revit “platform” which encompasses architecture, structure, and mechanical, electrical, and plumbing (MEP) domains.

Up-to-date software (e.g., Autodesk's Architecture, GraphiSoft's ArchiCAD, Bentley's Microstation, Tekla's Structures, etc.) supports input and output of IFC files toward interoperability. Software vendors advertise their products as “IFC-Compliant” to the latest version of IFCs. For instance, Revit Architecture can import IFC files from Revit Structure and vice versa. Tekla is notable in the structural engineering domain. Tekla Structures is similar to other BIM applications for structural design: instead of drawing 2D structural plans, sections, and elevations, structural engineers can create a complete digital model that simulates a real world structure and combines both the physical model and the analytical model. Since Tekla Structures is fully compliant to IFC2x3, a Tekla model can be exported as an IFC file and opened in other BIM applications, while exported IFC files from other applications can be used as reference models in Tekla Structures.

### 1.2. Problems and Limitations of Current BIM Technology

Eastman's and other centralized data models were well defined and tractable enough to support a limited number of participants. However, the complexity of the architectural product has generated more problems than the shared data model could solve. Current BIM suffers from a number of shortcomings which are as follows [13].

- In order to ensure as wide an agreement as possible within the industry on semantic definitions, it requires continuous and iterative review by the professionals. This is also the most time-consuming factor identified by Wix and Liebich [14].
- Since it relies on human review, it is error-prone to integration of all domain development into a single model and to make it internally self-consistent.
- Semantic definitions covering possible domains are inevitably at high level, which results in lack of defining details such as detailed ontological information through the design process and the construction.
- Captured models work only for a short period of time. It is almost impossible to add what a participant needs for a specific purpose because of static structures of the models.

Although the IFCs contain detailed information about construction projects, only a small portion of all the important data that can exist throughout any project element is represented [15]. The IFCs have a mechanism to accommodate information, that is, not explicitly modeled by defining property sets of additional object data. End users can define property sets in advance, based on an agreement between two software vendors or by representatives from a specialty group within the industry [15, 16]. Nonetheless, it would be difficult for end users to come up with property sets against problematic situations that might arise throughout the project life cycle.

The IFCs do not have specific rules about how their elements are created and organized. This is dependent on software itself in conjunction with its user interface. In order to maintain internal model consistency, Revit implements strong rules about how building elements are created, organized, and represented, which are not compatible with the IFCs. Thus, it would be impossible to expect a seamless file exchange between BIM applications even if they are all IFC compliant, unless they follow some sort of standards on how elements are created and mapped with respect to each other [17].

The primary objective of any centralized data model is to build a common data structure that would include all the possible representations using abstraction, hierarchy, and relationships to represent the building information. The notions of abstraction and hierarchy are useful for structuring a building description, as they allow each domain dealing with a building to add its own descriptions to it. However, it has become clear that a single, centralized data model would not serve all the requirements of all the participants [18–21]. In addition, the sheer magnitude of the combined data often exceeds the capability of its management by any one domain.

A new approach proposed by this research focuses on how the participants in the design process alternate between their “private” representations, which they use during their own, internal design process, and the “public” version which they “publish” for the benefit and use of the other participants. The core of this approach is a kind of “filtering” mechanism that mediates between the private (domain-specific) workspace and the shared public workspace.

The filter mechanism can be used for reflecting on the participants' design progress and for identifying potential problems in their performance as a self-evaluating tool. The filter-mediated communication model for collaborative design is proposed to reflect the characteristics of multidisciplinary collaborative design to facilitate participant-oriented aspects and to solve real world collaboration problems by focusing on a semantically rich representation method and distributed mode of communication which is mediated by intelligent filters. A similar distributed approach is found in “the engineering framework (TEF)” [22], an adapter-based solution for integrating plant engineering information. However, TEF does not deal with semantics of information.

To demonstrate the proposed model, a hypothetical case study is presented, which incorporates computational methods to support the process of knowledge creating, storing, and sharing among different professionals without sacrificing human aspects and to validate the feasibility of the model.

## 2. Filter-Mediated Communication Model

### 2.1. Concept of Filter-Mediated Communication

**Figure 1:**
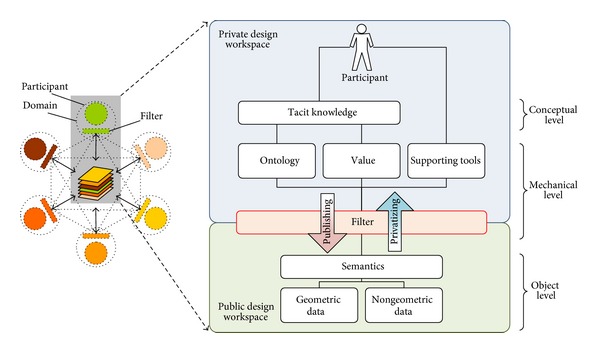
Anatomy of filter-mediated communication.

In order to overcome the limitations of semantic representations in the current BIM technology, we developed a “filtering” mechanism. As shown on Figure 1, individual participants have their own private workspace separate from the public workspace, that is, shared with all the participants. The filter's role is to manage design representations by alternating between these two different workspaces. The filter, as a software agent assigned to a specific participant, generates public versions from the participant's private design representations by removing notations, sketches, calculations, and other information that are unnecessary to other participants. The filter also serves the inverse mode to produce domain-specific, private versions of design representations by interpreting and translating public design data received from other participants.

Assume that two participants, P-A and P-B, collaborate in this setting. P-A can generate a variety of design solutions, which are unofficial, domain-specific, and semantically rich to P-A, in P-A's private workspace. When P-A decides to publish one of the design solutions, P-A's filter (F-A) transforms the selected design solution (privateVersion-A) into an official, domain-neutral version (publicVersion-A) and publishes it to the public workspace. Conversely, when P-A wants to review P-B's solution published in the public workspace, F-A transforms it (publicVersion-B) to be domain-specific and semantically rich to P-A (privateVersion-B) in P-A's private workspace. In these two contrary processes, the filter, F-A, changes modes (i.e., private or public) of design representation based on the P-A's predefined, domain-specific ontology.

The public workspace provides the participants with object level information that includes geometric (dimension, level, grid, etc.) and nongeometric data (cost, schedule, project, etc.) and their semantic data, whereas the private workspace contains the conceptual and the mechanical levels of information. The conceptual level stores participant's tacit knowledge, and the mechanical level consists of domain-specific ontologies and values and supporting tools. Furthermore, to enable a high-level communication among the participants, the filter serves both syntactic and semantic communication within the object level. The syntactic communication allows different applications to read the data and the semantic communication provides the ontological meanings associated with the data. The next section will describe the detailed characteristics of the filter-mediated communication.

### 2.2. Characteristics of Filter-Mediated Communication

#### 2.2.1. Semantic and Syntax Communication

**Figure 2:**
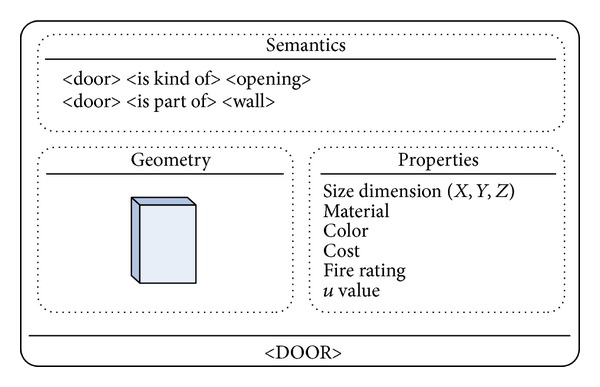
A “DOOR” ontology.

The semantic level is the first step towards interdomain communication. The conceptual ontology adds to the object data that any one domain expert may take for granted, but which would be viewed differently by another domain expert. It thus provides a more explicit but abstract way to describe information, encapsulating both conceptual and domain-specific data models. The conceptual models may include elements such as generalization, aggregation, and cardinality constraints about the objects (e.g., a door is a kind of opening and belongs to a specific wall) (Figure 2). The domain models deal with vocabularies defined by domain-specific ontologies, such as architecture, structural engineering, mechanical engineering, and general contractor to name a few.

This level fulfills the most important role in the filter model: adding semantically rich information that can be interpreted correctly by different domains of knowledge. As an example, consider the design of a house. The plans produced by the architect include objects such as walls, rooms, and openings. The structural engineer must interpret the plans, retrieving the meanings of the objects it contains. However, the structural engineer's interpretation is often different from the architect's: he uses different vocabularies, such as “bearing walls,” “partitions,” and “frames.” The structural engineer may, therefore, begin his task by translating the objects from the architect's representation into his own objects, creating a totally different representation of the same floor plan. The architect will have to go through a similar process when he receives the structural engineer's drawings.

The proposed filter mechanism will automate this interpretive translation process, using the semantics associated with each object that comprises the plans. It will interpret, add, or omit data as needed by the domain expert. For example, the structural engineer's filter will interpret architectural WALL objects as LOAD_BEARING or NON_LOAD_BEARING objects, without burdening the architect's representation of the same data. With the addition of the suggestion-based applications, the architect may be alerted when he tries to modify a wall designated LOAD_BEARING by the structural engineer, but the data describing the wall's specific load bearing properties will be hidden from him.

The syntax level is intended to provide a common standard for exchanging data. We propose to adopt extensible markup language (XML) as a common syntax. The main task of the syntax level, therefore, would be to tag each object using appropriate XML tagging. The tags alone will not carry meaning unless they are connected to ontological information which is stored in the semantic level.

#### 2.2.2. Peer-to-Peer Communication

In terms of computer networks, there are two common models of computer networking: the client-server model and the peer-to-peer model (P2P) [23]. In a client-server model, the client (the user's computer) makes requests of the server to which it is networked. The server, typically an unattended system in a back room, responds to and acts on the requests. Data-centric approaches usually use this model. On the other hand, the idea behind the P2P model is that each “peer,” that is, each participating computer, can act both as a client and as a server. The notion of decentralization is directly applicable to this model.

**Figure 3:**
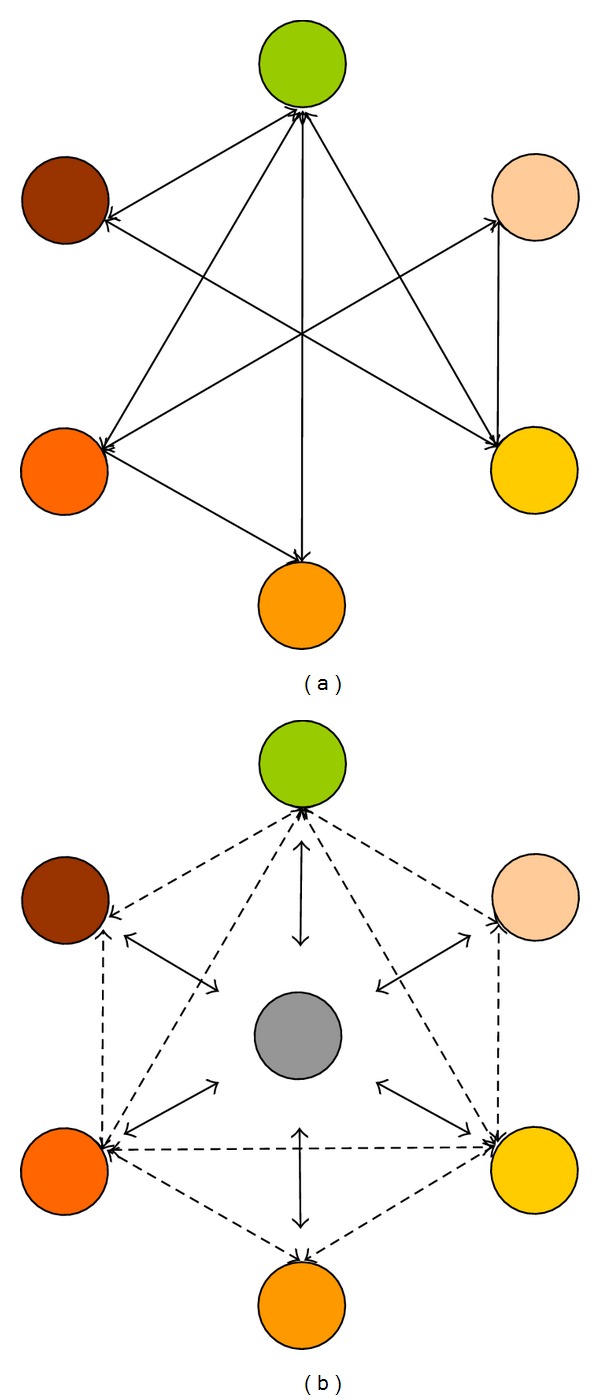
Two communication modes: (a) pure peer-to-peer and (b) hybrid peer-to-peer.

Pure P2P computing (Figure 3(a)) has no central server and router and peers in this network act as clients and servers. Hybrid P2P (Figure 3(b)) has a central server that keeps information on peers and responds to requests for that information. Peers are responsible for hosting the information, for letting the central server know what information they want to share, and for downloading its shareable resources to peers that request it.

The major advantage of the P2P model over the client-server model is that we can reduce the size and complexity of the centralized data and even eliminate centralized control over the data, which is expected to overcome the problems of the data-centric approach discussed earlier. For practical purposes, we adopt the hybrid P2P model for filter communication. This is because in the pure P2P model, peers may spend much time and effort to find other peers and their resources. The shared data includes general information on peers, agreements on how to describe ontological specifications for the specific domains, and the latest version of each participant's published data.

We regard each participant's filter as a peer in the filter communication. When the filter obtains a connection to the server which has the shared data, it examines the consistency of its maintained published data. First, it will publish the latest detected version of each participant's data. Second, if it detects inconsistency between the published data and the private data, it will update its published data. Since each participant's private data is dynamically changed in the course of the design, the filter will update its own published data when it is requested to provide information by other filters.

#### 2.2.3. Filter Mechanism in Design Process

The filter mechanism is intended to reduce ambiguity and clarify the choices made by each participant in the design process, without encumbering the participants through overprescriptiveness. Ambiguity in design process has two contradictory aspects: one is a powerful enabling force for creative design and multidisciplinary planning [24]. The other is an obstacle to achieving quality and can introduce cost deficiencies in construction such as inconsistent and contradictory client's specifications, ambiguity about organizational structure and responsibilities of the design team, and ambiguity in the design documents themselves [25]. With the conception that ambiguity plays different roles through the design process, we will discuss the role and function of the filter mechanism which will deal with ambiguity along the design process.

**Figure 4:**
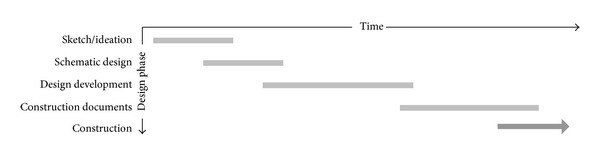
Typical phases in the design process.

The design process is generally considered to consist of four distinctive phases before actual construction [26]: (i) sketch/ideation, (ii) conceptual/schematic design, (iii) design development, and (iv) construction documents (Figure 4).

*(i) Sketch/Ideation*. In the early phase of design (sketch/ideation), ambiguity is desirable, where designers are brainstorming freely in order not to stifle design possibility too early. They interpret client's specifications and requirements as well as particular environmental settings identifying problems and solutions at the same time. At this point, opinions contributed by the designers are fairly scattered and are not likely coherent.

Even though there are a plethora of tools that can support various aspects of the design process, most of them (AutoCAD, MicroStation, VectorWorks, etc.) are focused on later phases (especially “construction documents”) of the design process. The reason is that the design problem in the early phase is usually ill-defined [27, 28], and designers are unable to evaluate the participants' contributions due to the incompleteness and incoherence of data.

Although there would be difficulty having concretized answers in this phase, this would be another opportunity to raise a variety of possible issues and problems and explore solutions before making irreversible decisions. The filter mechanism emphasizes a participant-oriented approach in that participants can enrich ontological information with their knowledge, belief, and specialized tools. Each participant can contribute his or her knowledge so that it can help other participants propose solutions and solve problems. For example, when an architect who has little knowledge about the requirement of a fire-egress door starts partial ontological information of the door, a fire marshal can contribute his or her knowledge to the door through the filter mechanism.

*(ii) Conceptual/Schematic Design and (iii) Design Development*. In the following phases, “conceptual/schematic design” and “design development,” ambiguity becomes less desirable. The design becomes more concrete, focused on specific design issues and problems (e.g., adjacencies, dimensions, materials, structure, views, orientations, etc.). The participants would need more information from a variety of sources in order to solve the design problems and evaluate proposed design alternatives based on multiple criteria. It is at this phase that tools that can support meaningful exchange of information become desirable.

**Figure 5:**
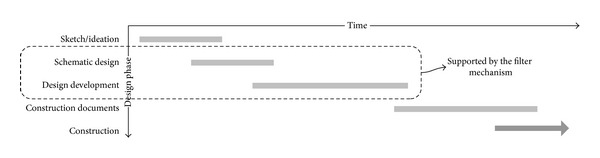
Filter mechanism in the design process.

The proposed model is intended to support these phases, where the most important design decisions that require multidisciplinary collaboration are made, where there is enough information contributed by the participants from different disciplines to make substantive design decisions and where the design has not yet been developed so much that changes are no longer feasible (Figure 5).

Surprisingly enough, there are very few frameworks or systems to support these phases of the design process. One of the reasons is that “over-the-wall” practice (serial approach) is still pervasive in the AEC industry so that it is difficult for a discipline to predict the impact of its decision on another's. However, the impact of a network-based collaborative design transforms a hierarchical/linear partitioned process into a distributed and interleaved one. Using the filter-mediated communication model, the participating professionals can affect one another bi- or multidirectionally.

*(iv) Construction Documents*. “Construction documents” is the final phase where the design is more constrained and structured, where ambiguity should be avoided, where the detailed designs are made and documented for construction and where project management is heavily involved. Since most major design decision-makings have been made, the participants exert their efforts on documentation and dissemination of the design. In the last decade, the AEC industry has seen the development of web-based project management applications (also called project portals) which aim particularly to support that purpose (e.g., AutoDesk's Buzzsaw, Bentley's ProjectWise, etc.) [29].

Even though the proposed model is not aimed at this phase, the participants would benefit from the model in that they would better understand the necessary kinds of information and representations for actual construction.

## 3. Implementation of Filter-Mediated Communication Model

### 3.1. Modeling of Building Ontology

**Figure 6:**
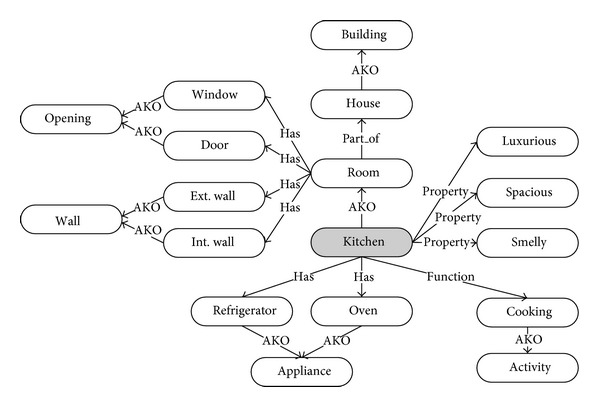
A kitchen ontology with semantic network.

In order to model a building ontology, we applied a semantic network [30] that includes nodes to represent concepts and links to define interconnectedness between nodes. As shown on Figure 6, the links can be labeled differently such as “A_KIND_OF,” “PART_OF,” and “HAS_A” to describe inheritance, hierarchy, and assemblage, respectively.

Because the semantic network has a flexible structure, unlike other building models (STEP, IFC, EDM, etc.) that have a rigid and static structure, it can contain an infinite number of descriptions of building components within the network according to participants' ontological information [31, 32]. However, despite its flexibility, participants need to define a minimum set of ontological information that agreed with other participants for communicational and collaborative efficiency.

**Figure 7:**
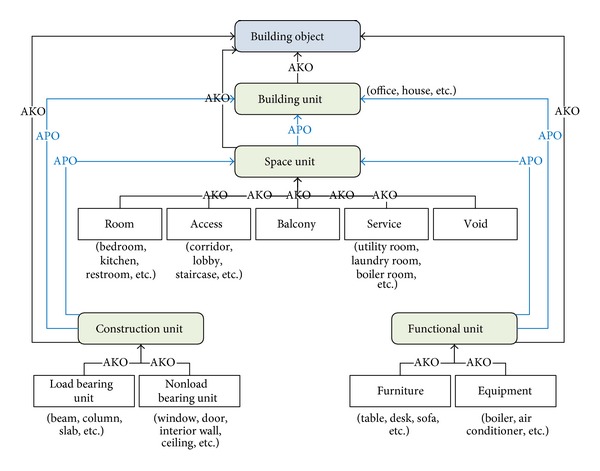
Structure of building object ontology.

Figure 7 shows the overall structure of the building ontology which consists of four common ontological units: building unit (BU) (house, office, school, etc.), space unit (SU) (room, bedroom, bathroom, etc.), construction unit (CU) (slab, floor, wall, etc.), and functional unit (FU) (furniture, equipment, etc.).

The four common ontological units (i.e., building, space, construction, and functional units) inherit the root object, “building object.” Conceptually, it is possible to define unlimited relationships, but there are only two relationships in the current model: A_KIND_OF (or AKO) and A_PART_OF (or APO). AKO means the subsumption relation and APO the assembly between two different units (or classes). Each of these four units can have many subnetworks with either AKO or APO. APO is the main relation between a Building Unit and the other three units to define assembly relationship.

SUs define architectural spaces and are defined by other physical entities such as CUs and FUs. Therefore, in the model, CUs and FUs are connected to SUs with an APO relationship. CUs are physical entities that comprise SUs. CUs are divided into two groups by their structural characteristic: load bearing and nonload bearing units. FUs include furniture and equipment that serve specific functional roles by occupying SUs.

### 3.2. Filter Implementation

In the filter-mediated communication, each participant has his or her domain-specific filter. During communication, the major role of the filter is to translate incoming design representations from the shared workspace and to publish outgoing representations based on the participant's ontology. For the flexibility and efficiency of implementing the domain-specific filters, Extensible 3D (X3D) [33] and XML Path Language (XPath) [34] were applied.

**Figure 8:**
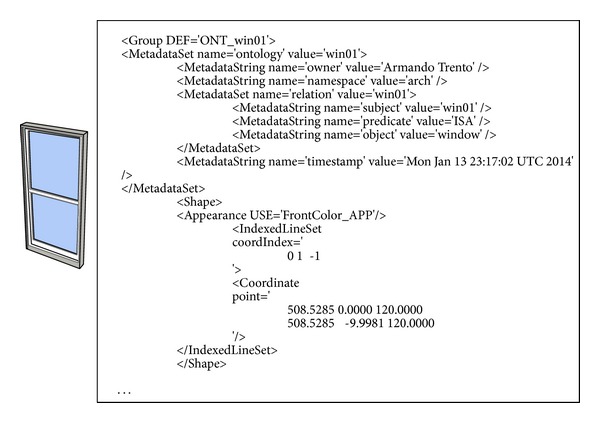
Window ontology in X3D.

In order to define ontological information in X3D, we incorporated a shorthand non-XML serialization of resource description framework (RDF) [35] considering its simplicity, neutrality, and extensibility. RDF expresses a relationship by connecting a subject and an object with a predicate.  Figure 8 represents a window with ontological information in X3D.

### 3.3. Workspace Implementation

**Figure 9:**
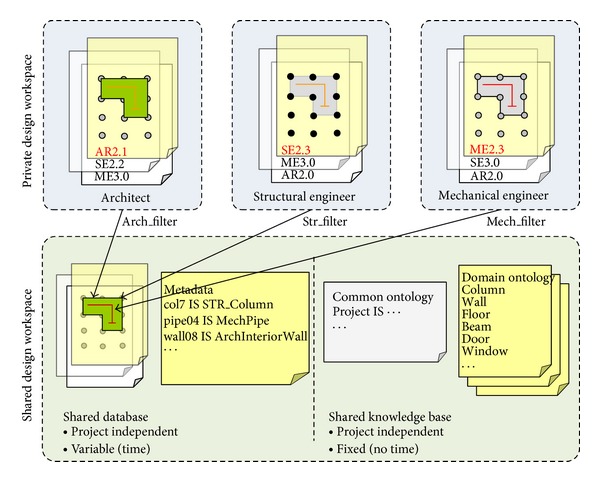
SDW and PDWs for three participants.

As described in the previous section, the filter mediates two different design workspaces: the private design workspace (PDW) and the shared design workspace (SDW). The PDW is an independent working environment exclusive to a specific participant. In the PDW, as a design progresses, the participant creates objects of which semantics are built with his or her domain-specific ontology. On the contrary, the SDW is a public environment open to all the participants and consists of a “shared database” and a “shared knowledge base.” The shared database contains project-dependent, ongoing design data contributed by the participants whereas the shared knowledge base contains project-independent, reusable ontology either common or domain-specific (Figure 9).

To be easily processed by the filters, XML, an application-neutral format, is used to store geometric and ontological data (in X3D) and query specific data (with XPath) in the SDW. With the shared data repository in XML, the filters can query domain-specific design representations and share them with other filters.

**Figure 10:**
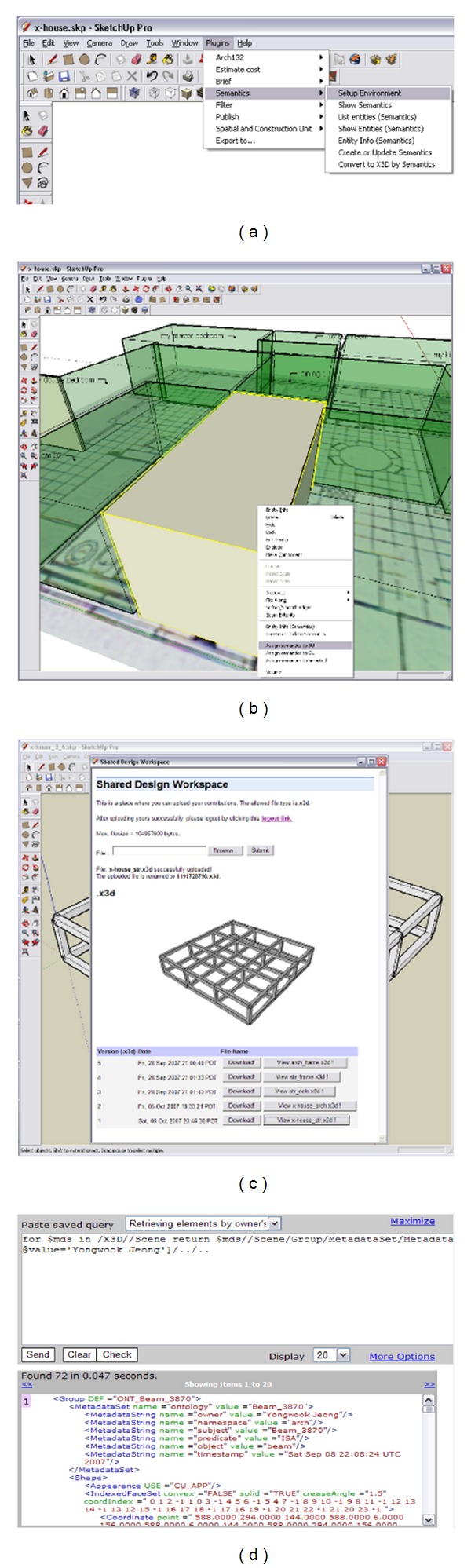
Screenshots of PDW and SDW: (a) PDW setting UI, (b) modeling in PDW, (c) design review in SDW, and (d) X3D query sandbox.

As shown on Figure 10(a), we developed several plugins and incorporated them into SketchUp for the participant to embed semantic information to objects designed in the PDW. The SDW has two components: a storage (Figure 10(c)), to which the participants upload public versions of design representations through their filter, and an X3D query sandbox (Figure 10(d)) for the filter to extract design information from the SDW.

**Figure 11:**
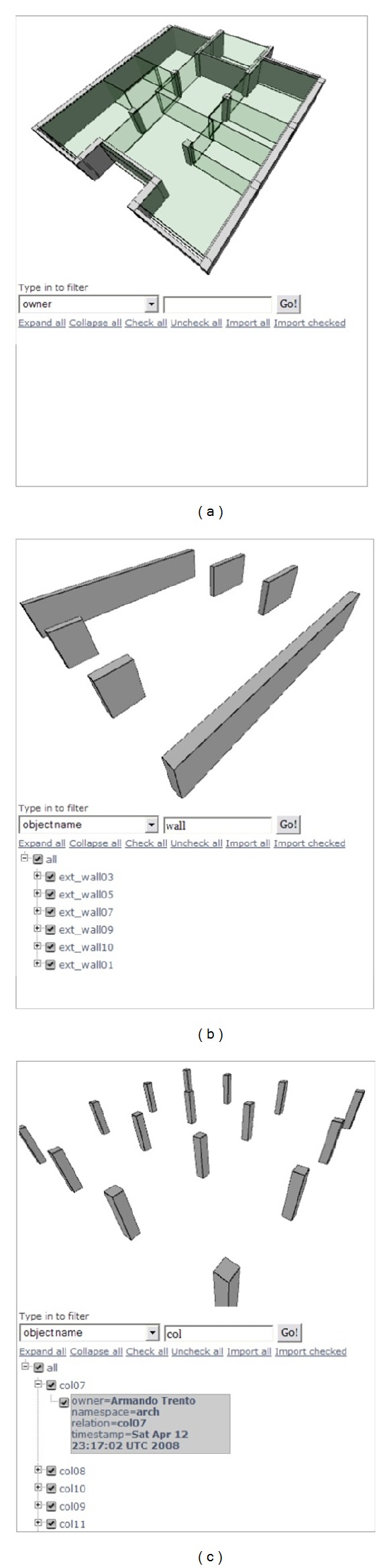
Filtered representations of objects.

Suppose a 3D plan of an office that has multiple SUs and CUs by the architect as shown in Figure 11(a). The model is in the X3D format and uploaded to the SDW so that the XML database will manage it. When the structural engineer comes in and wants to see CUs only, he or she can type in texts that might be relevant domain ontology. The filter creates appropriate queries against the model in XPath and sends them to the SDW to get any result.  Figure 11(b) shows a result by typing “wall” in the blank. The queries are executed against the stored representations by the XML database, and the results are sent back to the structural engineer's filter. The structural engineer's filter displays the result in the order of queried names shown in Figure 11(c).

## 4. Case Study: Filter-Mediated Collaborative Design

**Figure 12:**
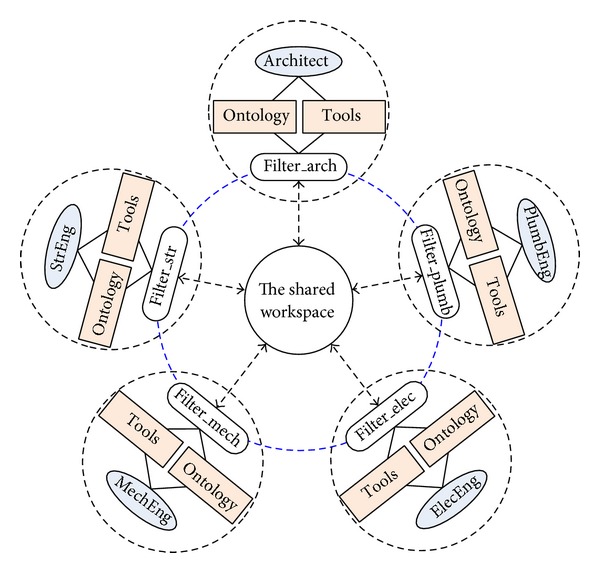
A hypothetical setting for the office building project.

This hypothetical case study illustrates the function and process of the filters in a multidisciplinary collaborative design environment: the core and shell design of an office building (Figure 12). The office project has a group of different participants including an architect, a structural engineer, a mechanical engineer, an electrical engineer, and a plumbing engineer.

First, in order to simplify the process, the owner is assumed to specify his or her requirements that will be shared by all the participants. Interpreting the owner's requirements and desires, the architect generates some schematic designs that roughly meet the owner's needs. At this stage, the role of the architect would be that of a consultant who helps the owner realize their vision concretely. If the owner is satisfied with the architect's proposed design, the architect begins developing a design. In the course of the design, the architect may encounter several issues that must be resolved in collaboration with the other participants. In a similar way, the other participants may have similar conflicts that need to be resolved.

Since they have different knowledge, representations, and their own discipline tools, the participants are subject to interpret the input data in their own unique ways. In this case study, we focus on the information flow among the participants: what information is transmitted and how each filter interacts with another collaborating participant. The basic assumptions for this case are as follows.

- Each participant deals with one aspect of a whole design.
- Each participant is responsible for creating his or her own ontological information.
- Each application has its own data model that cannot be directly read by other applications.
- The published data is written in XML, which can be processed by all the participants.
- Each participant is in charge of retrieving the data from the shared workspace when another participant publishes data.

### 4.1. Participants in Collaborative Design Process

#### 4.1.1. Architect

The architect is in charge of creating a schematic design while considering the project program and any particular design criteria specified by the owner. The architect's primary interest is spatial quality, and thus he might start his design with defining spaces according to the requirements, design criteria, and so on. An SU can be defined by a number of CUs as discussed.

**Figure 13:**
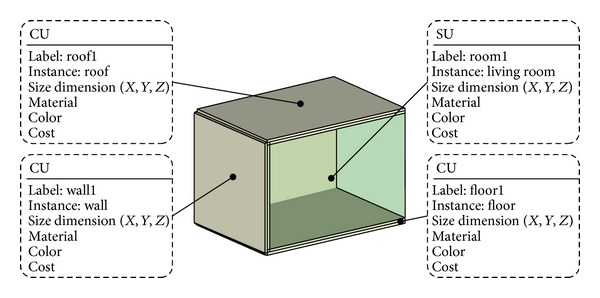
A room with the architect's ontology.

Before creating drawings, he starts with defining his ontologies. Although he can reuse some ontologies independent of a specific project (e.g., individual products including doors, windows, etc.), he has to define particular ontologies if necessary. For example, he draws a box with dimensions (geometric information) and properties (nongeometric information) which are dependent on a specific project. Then, defining it as a “wall,” a “floor,” a “roof,” and so on, he can build a new ontology (Figure 13). In this case study, he creates a 3D model as a common denominator and his filter will publish it in XML to the SDW.

#### 4.1.2. Structural Engineer

**Figure 14:**
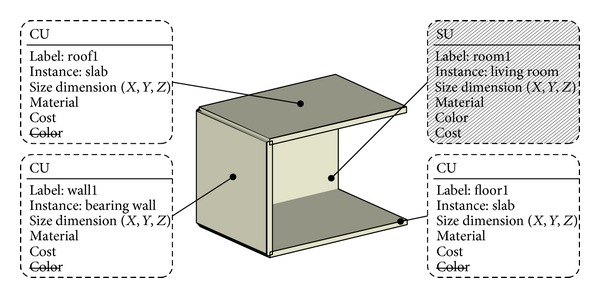
Enriched representation by the structural engineer's filter.

In order to design the structure, the structural engineer needs the architect's models as well as structural codes and standards. Since the input does not have information on structural analysis, the structural engineer's filter will rebuild the model based on his own ontologies. For example, when the structural engineer's filter receives the architect's model that was designed schematically, it tries to differentiate the model to generate proper representation for structural analysis (Figure 14).

The structural engineer's primary concern is how the architect defines his spatial definitions and how the definitions are constructed by CUs. Although the architect might use the same labels, such as “wall” and “floor,” the structural engineer might have different definitions. Thus, the structural engineer can choose relevant ontological definitions that the architect has defined. Additionally, based on the structural engineer's ontology, his filter ignores irrelevant information such as color, that is, not important for structural calculation. If the architect's ontology does not have enough definitions, then the structural engineer can add his own ontological information such as “inertia” and “moment” properties to the model. Based on this information, the structural engineer will do structural analysis using his own disciplinary tools. If he is ready to publish his design, his filter will publish it to the SDW so that the other participants' filter can access it.

#### 4.1.3. Mechanical/Electrical/Plumbing Engineer

The mechanical systems in this case are HVAC supply ducts. The plumbing engineer's task is to route sanitary waste, and the electrical engineer has to deal with cable trays and conduits. The MEP engineer's primary concern is whether the corridor ceiling spaces are deep and wide enough to contain the necessary MEP systems. Therefore, the architect's and structural engineer's design criteria usually act as constraints.

**Figure 15:**
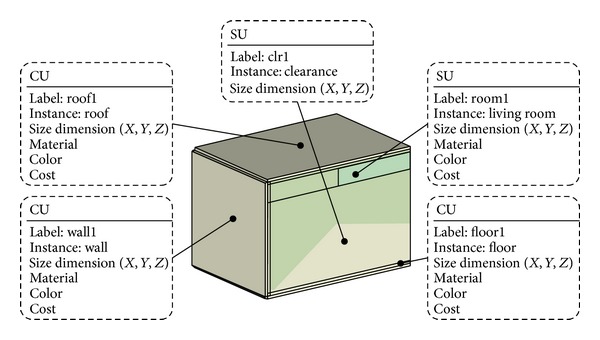
Enriched representation by the MEP engineers' filters.

The filters collaborating with the MEP engineers will induce clearance and available spaces from the input geometry (Figure 15). Then, the filters will ignore material, color, cost, and rigidity and add “clearance” to the model. The electrical engineer's task is even more complicated because his design has to harmonize with the layout design of other MEP systems while complying with constraints imposed by architectural design and clearances required by code and specification.

### 4.2. Filter Operations in Design Process

**Figure 16:**
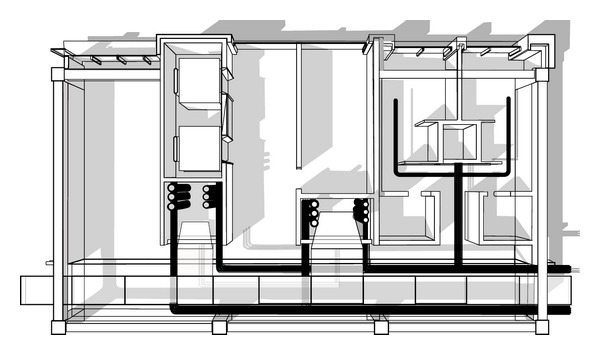
The core of an office building.

Figure 16 shows a partial plan of an office building where the participants collaborate with each other. The objectives of this section are:

- Dynamic interaction among participants (producers/consumers of information),
- Ontological information flow (knowledge representation),
- The role of the filter in a design process:
  - Intelligent filter to decode/encode the published data,
  - Enriching input data based on ontologies of a participant,
  - Suggestion mechanism.

The process in Table 1 describes only a part of the rather lengthy and iterative design development process. The process envisions that each participant uses their own knowledge and representational methods, and that their intelligent filter retrieves other participants' knowledge in order to interpret their own representations. It also explains that an object can be understood from within more than one domain at the same time, thereby raising the possibility for multiple interpretations. Through the filter-mediated communication model and the process, the participants are expected to save time and effort in that they would know the implication of their actions ahead of time.

## 5. Discussion

Recently, to tackle semantic issues in IFC based information exchange, “information delivery manual (IDM)” and “model view definition (MVD)” have been introduced [36–38]. Their major objective is to enhance interoperability between IFC-compatible software applications by providing technical specifications. Because they mainly focus on syntactical interoperability between applications, they may not fully support domain-specific and semantically rich design representations of diverse participants. In this study, we propose a methodology that can reflect the essence of design collaboration, which inevitably characterizes short iterations and frequent changes. Through the filter-mediated communication based on ontology, the participant is able to choose the most relevant model (e.g., IFC), definition methodology (e.g., MVD), and deployment (e.g., SDW in this study) for enhancing shared understanding.

As the case study is to verify the conceptual feasibility of the proposed model, there are limitations that need to be considered for practical application. First, because the ontology itself does not have a mechanism to detect any duplicity between different ontologies, to maintain ontological consistency without semantic conflicts, the participants need to agree on their ontologies in advance by, for example, sharing a glossary. Second, design dependency, which is an important aspect in design collaboration, has to be considered. Dependencies between participants' design solutions can be managed by specifying sender and receiver data. Moreover, through design collaboration, the participants' various design versions should be controlled in some way in order to avoid conflicts among outdated versions. One possible way is to use some popular version control system in the field of programming, such as concurrent versions system (CVS) [39] or subversion [40] assuming that ontologies are created in a machine-processable format (e.g., XML). Lastly, an unobtrusive way to create the participants' ontologies should be prepared for sharing them with ease considering user experience aspect. We will delve into issues related to these limitations in our future works.

## 6. Conclusion

In this paper, we have discussed the concept and implementation of a filter-mediated communication model for collaborative design in order to try to answer the following questions.

- How could knowledge be represented for collaborative design?
- How could it be shared (or communicated) for that purpose?
- How could it be queried?

To answer the first two questions, we propose the filter-mediated communication model and its process to reflect the characteristics of multidisciplinary collaborative design by focusing on a semantically rich representational method based on ontology that is mediated by intelligent filters. By fulfilling the discussed tasks, it is expected that the designers from different disciplines for an AEC project can better understand the dynamic process of collaborative design for achieving a high level of shared understanding.

We also present a computational implementation of the filter mechanism. It assumes that there are two distinctive design workspaces (i.e., the SDW and the PDW) facilitated by the filter operated by the individual participants. The model uses XML as an underlying technology that enables integrating semantics into geometries. A possible implementation is presented through off-the-shelf applications (i.e., SketchUp and its customization language). The participants benefit from the filter-mediated communication in that they can retrieve relevant representations from their SDW by creating user-definable queries (from simple filtering based on keywords to enriching representations through in- and outpublish. One participant also can see others' perspectives by adopting their filter, which would be an answer to the third question.

Through the proposed filter-mediated communication, we expect to achieve the following benefits: (i) participant-oriented representation, (ii) distributed, (iii) interleaved communication, and semantics-aware document management. First, once the participants contribute their knowledge to the representation, the filter can make each participant see the other's point of view. The dynamic and semantically rich representation would allow the participants to make alternatives reflecting their intents more effectively, which eventually would lead to a state of shared understanding. Second, the impact of a network-based design collaboration can transform a hierarchical, linear partitioned process into a distributed and interleaved one. As a result, the participants do not have to share a large and heavy integrated model. Rather, their intelligent filter will access the information in the object level (geometric/nongeometric information and ontologies) which resides at its own location and translate it into their own representation using user-defined ontologies. This can fill the gap between the heterogeneous representations while preserving semantics. Lastly, when an object appears in more than one data set, with different ontologies (e.g., the architect's wall and the structural engineer's wall), the filter can recognize this duplicity and perform consistency-management, possibly formulating queries on behalf of a participant. Through this process, the participant may reduce design errors and delivery time without sending his or her designs to other participants and achieve the design goal more efficiently.

## Figures and Tables

**Table 1:** Filter-based collaborative design process.